# The differing influence of several factors on the development of fatty liver with elevation of liver enzymes between genders with metabolic syndrome: A cross-sectional study

**DOI:** 10.1371/journal.pone.0177925

**Published:** 2017-06-02

**Authors:** Masahiro Sogabe, Toshiya Okahisa, Masahiko Nakasono, Hiroshi Fukuno, Yoshihiko Miyamoto, Yasuyuki Okada, Jun Okazaki, Jinsei Miyoshi, Tetsu Tomonari, Tatsuya Taniguchi, Takahiro Goji, Shinji Kitamura, Hiroshi Miyamoto, Naoki Muguruma, Tetsuji Takayama

**Affiliations:** 1 Department of Gastroenterology and Oncology, Institute of Biomedical Sciences, Tokushima University Graduate School, Tokushima, Japan; 2 Department of Gastroenterology, Kagawa Prefectural Cancer Detection Center, Takamatsu, Japan; 3 Department of Internal Medicine, Tsurugi Municipal Handa Hospital, Tokushima, Japan; 4 Department of Internal Medicine, Higashi Tokushima Medical Center, National Hospital Organization, Tokushima, Japan; INRA, FRANCE

## Abstract

**Background:**

Nonalcoholic fatty liver disease (NAFLD) is known to be strongly associated with obesity, visceral fat, metabolic syndrome (MS), lifestyle, and lifestyle-related diseases in both males and females. However, the prevalence of NAFLD, MS, and clinical backgrounds is different between males and females.

**Objective:**

We conducted a cross-sectional study to examine the differing influence of lifestyle-related factors and visceral fat on fatty liver (FL) with elevation of liver enzymes between males and females with MS.

**Methods:**

We enrolled 42,134 persons who underwent a regular health check-up, and after excluding subjects who fulfilled excluding criteria, the remaining subjects were 2,110 persons with MS. We examined the differing influence of lifestyle-related factors and visceral fat on FL with elevation of alanine aminotransferase (ALT) (ALT elevation was defined as ALT level of ≥31 IU/l in the present study).

**Results:**

The odds rations for FL with ALT elevation were as follows: WC, 1.83 (95% confidence interval (CI) 1.36–2.46); dyslipidemia, 1.89 (95% CI 1.34–2.68); hemoglobin A1c, 1.36 (95% CI 1.00–1.85); visceral fat type MS (V-type MS), 5.78 (95% CI 4.29–7.80); and light drinker, 0.56 (95% CI 0.41–0.78) in males with MS and BMI, 2.18 (95% CI 1.43–3.33); WC, 1.85 (95% CI 1.27–2.70); diastolic blood pressure, 1.69 (95% CI 1.16–2.45); triglyceride, 2.22 (95% CI 1.56–3.17); impaired glucose tolerance, 1.66 (95% CI 1.11–2.47); and V-type MS, 3.83 (95% CI 2.57–5.70) in females with MS. The prevalence of FL with ALT elevation and ALT was significantly higher in V-type MS than in the subcutaneous fat type MS in both males and females with MS (P < 0.001).

**Conclusion:**

Although V-type MS and WC is a common significant predictor of an increased prevalence of FL with ALT elevation in both males and females with MS, gender, lifestyle-related factors, and MS type in individuals with MS should be considered for the development of FL with ALT elevation.

## Introduction

Metabolic syndrome (MS) is commonly defined as the presence of an increased waist circumference (WC) and more than two of the following components: (i) high blood pressure and/or medication for hypertension; (ii) hypertriglyceridemia, low levels of high-density lipoprotein (HDL) cholesterol and/or medication for dyslipidemia; and (iii) hyperglycemia and/or medication for diabetes [1, 2]. In other words, MS is recognized as visceral fat with lifestyle-related diseases. There has been a recent increase in the rate of individuals with MS in both developed and developing countries due to economic growth and changes in lifestyle such as insufficient physical exercise and excess food consumption [3]. It is now becoming accepted that MS is an important issue concerning public health and medical expenses. MS has been highlighted as a risk factor for ischemic heart disease and arteriosclerotic diseases, and has recently been shown to be strongly associated with non-alcoholic fatty liver disease (NAFLD). Although MS is associated with NAFLD in both sexes, the prevalence of MS and NAFLD is known to differ between genders. In addition, several factors associated with MS, such as the distribution of adipose tissue, lifestyle, and the prevalence of lifestyle-related diseases, also differ between genders. In this study, we elucidated the differing influence of lifestyle, lifestyle-related diseases, and visceral fat on fatty liver (FL) with elevation of liver enzymes between genders with MS.

## Methods

### Subjects and study design

Forty two thousands one hundred thirty four Japanese adult subjects who underwent a regular health check-up at our hospital between April 2008 and March 2013 were initially included in this study. The exclusion criteria were as follows: (1) subjects who were positive for hepatitis B surface antigen (HBsAg) and/or hepatitis C antibody (HCVAb); (2) subjects who did not fulfill the diagnostic criteria for MS; (3) subjects who did not undergo an ultrasound; (4) male subjects with an alcohol consumption of 20 g/day or more; (5) female subjects with an alcohol consumption of 10 g/day or more; (6) subjects who had a history of liver surgery; and (7) subjects who take and/or took medicine for liver disease. The regular health check-up included taking a record of the previous medical history, present medical condition, conducting physical examination, and routine biochemical variables. Body weight and height of the subjects were determined, and body mass index (BMI) was calculated. WC was measured at the umbilical level. The measurements of blood pressure was performed more than twice, while the subjects were seated before collecting of blood samples. Venous blood samples were taken from all subjects in the morning, after the subjects had fasted for more than 12 h overnight. The levels of alanine aminotransferase (ALT), aspartate aminotransferase (AST), γ-glutamyl transpeptidase (GGT), total cholesterol (T-CHO), triglyceride (TG), HDL cholesterol (HDL-C), low-density lipoprotein cholesterol (LDL-C), uric acid (UA), fasting plasma glucose (FPG), and hemoglobin A1c (HbA1c) were determined by common enzymatic methods using an auto analyzer (TBA-80FR; Toshiba Medical System, Tokyo, Japan). All subjects were informed that the clinical data obtained by the medical check-up may be retrospectively analyzed, and written informed consent was obtained. The present study was a retrospective cross-sectional study clarifying the different influence of several factors on FL with ALT elevation between genders with MS. The study design was approved by the Ethics Committees of Kagawa Prefectural Cancer Detection Center, and the study was performed in accordance with the Declaration of Helsinki.

### Questionnaire for lifestyle factors

Lifestyle-related information, including alcohol consumption, smoking status, and medical history, were surveyed using a common standardized self-response questionnaire. Subjects were divided into two categories based upon the smoking information: never smokers, who had never smoked (Brinkman index (BI) [4] is zero); and smokers, who had a past history of smoking (BI is more than one). The amount of alcohol consumed per drinking day was determined in grams using representative percent alcohol by volume for each type of alcohol. Based upon the drinking information, subjects were divided into two categories: non-drinkers, males drinking 12 drinks or less per year of less than 20 g/drinking day, or females drinking 12 drinks or less per year of less than 10 g/drinking day; and light drinkers, males drinking more than zero but less than 20 g/drinking day, or females drinking more than zero but less than 10 g/drinking day.

### Diagnosis of MS

The diagnostic criteria for MS adopted by International Diabetes Federation (IDF) was used in this study [2]. Component factors of MS criteria are WC must exceed 85 cm for males or 80 cm for females, and the presence of two or more of the following: (1) dyslipidemia: HDL-C < 40 mg/dl for males, HDL-C < 50 mg/dl for females, and/or TG ≥150 mg/dl, or medication for dyslipidemia; (2) impaired glucose tolerance (IGT): FPG ≥100 mg/dl or medication for diabetes; (3) hypertension: blood pressure ≥130/85 mmHg or medication for hypertension.

### Assessment of ultrasonography

Abdominal ultrasonography was performed with the subjects in a morning fasting state. An Xario SSA-660A instrument with a 3.5 MHz convex-array probe (Toshiba Medical System, Tokyo, Japan) was used for ultrasonography. The criteria of FL diagnosis by ultrasonography were as follows: the FL must have liver-kidney echo contrast and liver brightness, as well as having liver vessel blurring and/or deep attenuation [5, 6]. Although liver biopsy is the gold standard for the diagnosis of NAFLD/nonalcoholic steatohepatitis (NASH), it is invasive and not realistic to perform in a medical check-up. Thus, we investigated FL with ALT elevation (ALT elevation was defined as ALT level of ≥31 IU/l) in the present study. In addition, assessment of visceral fat was performed using the abdominal wall fat index (AFI) that was defined by the ratio of the maximal preperitoneal thickness and the subcutaneous fat thicknesses at the same time point, and had a significant correlation with the ratio of visceral and subcutaneous fat areas by computed tomography (CT) [7]. The subcutaneous fat thickness was measured as the distance from the skin to the linea alba and the preperitoneal fat thickness was measured as the distance from the linea alba to the anterior surface of the liver. We defined AFI of at least 1 and AFI of less than 1 were regarded as V-type MS (in other words, dominant visceral type MS) and S-type MS (in other words, dominant subcutaneous type MS), respectively.

### Statistical analysis

Baseline data are expressed as the means ± standard deviation (SD). A *P* value of less than 0.05 was considered to be significant. Comparison of the proportion and the categorical variables between two groups was performed using the χ^2^-test. One-way analysis of variance was used to analyze the differences in baseline characteristics, such as age, physical examination, and serum biochemistry, between two groups. Factors with a significant influence on the prevalence of FL with ALT elevation were determined by univariate analysis. Age, BMI, WC, and factors that had a *P* value of less than 0.05 by univariate analysis were then subjected to a multivariate logistic regression analysis. The odds ratio (OR) and 95% confidence interval (CI) were analyzed for each variable. All statistical analyses were performed using Med Calc Software (Broekstraat, Mariakerke, Belgium).

## Results

### Enrollment of subject

**Fig 1:**
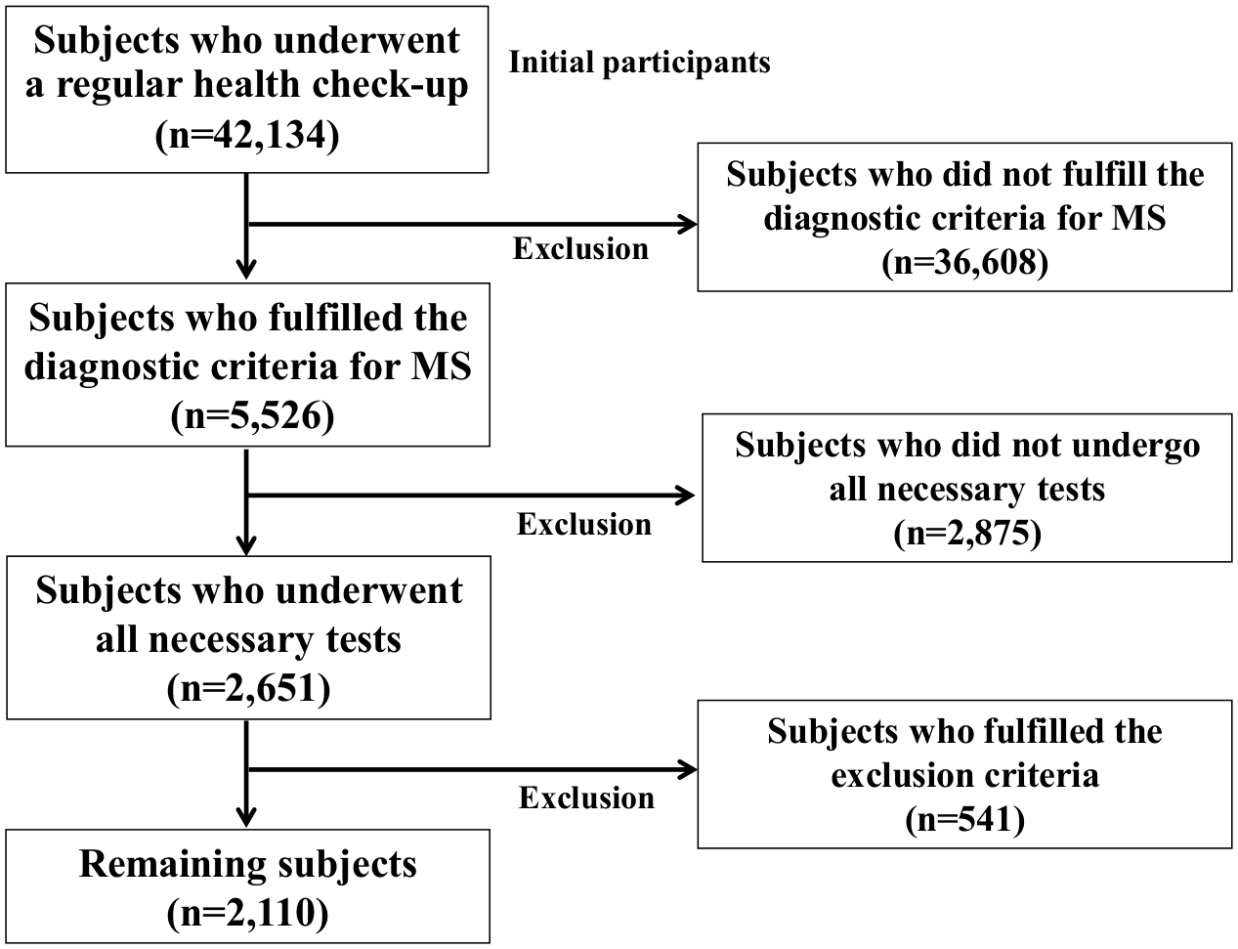
Flow chart of subjects selection. MS, metabolic syndrome.

The flowchart of the enrollment of subjects in the present study is shown in Fig 1. Forty two thousands one hundred thirty four subjects underwent a regular health check-up between April 2008 and March 2013 at our hospital. The enrolled subjects were selected by the inclusion and exclusion criteria described in the method section (subjects and study design). Of the initial 42,134 subjects, 5,526 (13.1%) subjects fulfilled the diagnostic criteria for MS. Of the 5,526 subjects, 2,651 subjects underwent all of the necessary tests for inclusion in the present study including physical examinations, blood-test screening, abdominal ultrasonography, and a self-response questionnaire. Of the 2,651 subjects, 541 fulfilled the exclusion criteria and were excluded, and the remaining 2,110 subjects were enrolled in the present study.

### Baseline characteristics of the subjects with MS

Baseline characteristics of the 2,110 enrolled subjects with MS are shown in Table 1. The proportion of males and females was 50.4% (1,063 males), and 49.6% (1,047 females), respectively. Mean age, mean BMI, and mean WC was 55.1 ± 9.2 years, 27.3 ± 3.6 kg/m^2^, and 93.3 ± 7.8 cm, respectively in all subjects. The prevalence of hypertension, dyslipidemia, and IGT was 78.0%, 83.6%, and 78.7%, respectively. The proportion of subjects reported to have alcohol consumption and a past history of smoking was 40.4% and 59.6%, respectively.

**Table 1:** Subject characteristics.

**Number**	**2110**
**Gender (male/female) (% male)**	**1063/1047 (50.4%)**
**Age (years)**	**55.1 ± 9.2 (21–85)**
**BMI (kg/m**^**2**^**)**	**27.3 ± 3.6 (18.5–50.8)**
**WC (cm)**	**93.3 ± 7.8 (80–141)**
**SBP (mmHg)**	**130.3 ± 14.7 (81–198)**
**DBP (mmHg)**	**78.9 ± 9.9 (45–127)**
**Hypertension (+/-) (% positive)**	**1645/465 (78.0%)**
**T-CHO (mg/dl)**	**208.2 ± 34.8 (93–380)**
**TG (mg/dl)**	**150.1 ± 91.6 (29–1353)**
**HDL (mg/dl)**	**54.3 ± 13.4 (26–127)**
**LDL (mg/dl)**	**134.5 ± 31.1 (48–286)**
**Dyslipidemia (+/-) (% positive)**	**1765/345 (83.6%)**
**UA (mg/dl)**	**5.7 ± 1.4 (1–10.9)**
**FPG (mg/dl)**	**111.1 ± 21.2 (65–272)**
**HbA1c (%)**	**6.1 ± 0.8 (4.3–12)**
**IGT (+/-) (% positive)**	**1660/450 (78.7%)**
**ALT (IU/l)**	**34.3 ± 24.6 (6–228)**
**AST (IU/l)**	**26.1 ± 12.9 (9–153)**
**GGT (IU/l)**	**51.0 ± 54.4 (7–663)**
**FIB-4 index**	**1.16 ± 0.66 (0.24–9.56)**
**Fatty liver (+/-) (% positive)**	**1401/709 (66.4%)**
**V-type MS / S-type MS (% V-type MS)**	**811/1299 (38.4%)**
**Alcohol consumption (+/-) (% positive)**	**1034/1076 (40.4%)**
**Past history of smoking (+/-) (% positive)**	**853/1257 (59.6%)**

Data represent the mean ± standard deviation (SD).

ALT, alanine aminotransferase; AST, aspartate aminotransferase; BMI, body mass index; DBP, diastolic blood pressure; FPG, fasting plasma glucose; GGT, gamma-glutamyl transpeptidase; HbA1c, hemoglobin A1c; HDL, high-density lipoprotein; IGT, impaired glucose tolerance; LDL, low-density lipoprotein; MS, metabolic syndrome; SBP: systolic blood pressure; S-type, subcutaneous type; T-CHO, total cholesterol; TG, triglyceride; UA, uric acid; V-type, visceral type; WC, waist circumference.

### Comparison of clinical characteristics between males with MS and females with MS

As shown in Table 2, BMI and WC were significantly higher in males with MS than in females with MS (*p* <0.05). The prevalence of hypertension, dyslipidemia, and IGT was 74.6%, 74.1%, and 83.1% in males with MS and 81.4%, 93.3%, and 74.2% in females with MS, respectively. The prevalence of dyslipidemia was significantly higher in females with MS than in males with MS (*p*^*a*^ <0.001). The prevalence of FL, ALT, AST, and GGT were significantly higher in males with MS than in females with MS (*p*^*a*^ <0.001). The proportion of V-type MS in males with MS and females with MS was 61.8% and 14.7%, respectively, and the prevalence of V-type MS was significantly higher in males with MS than in females with MS (*p*^*a*^ <0.001). The proportion of alcohol consumption in males with MS and females with MS was 73.8% and 23.9%, respectively. The proportion of past history of smoking in males with MS and females with MS was 72.5% and 7.8%, respectively. The prevalence of subjects who have alcohol consumption or past history of smoking was significantly higher in males with MS than in females with MS (*p* <0.001).

**Table 2:** Comparison of clinical characteristics between males with MS and females with MS.

	Males with MS	Females with MS	*P*-value
	(n = 1063)	(n = 1047)	(*P*^a^-value)
**Age (years)**	**53.3 ± 9.7 (21–83)**	**56.8 ± 8.3 (29–85)**	**<0.05**
**BMI (kg/m**^**2**^**)**	**28.2 ± 3.4 (21.6–49.3)**	**26.3 ± 3.5 (18.5–50.8)**	**<0.05**
**WC (cm)**	**96.6 ± 7.1 (90–141)**	**90.0 ± 7.1 (80–117.5)**	**<0.05**
**SBP (mmHg)**	**129.6 ± 15.0 (81–198)**	**131.0 ± 14.2 (83–192)**	**(NS)**
**DBP (mmHg)**	**80.0 ± 10.2 (46–127)**	**77.8 ± 9.5 (45–114)**	**(<0.001)**
**Hypertension, *n* (%)**	**793 (74.6%)**	**852 (81.4%)**	**(NS)**
**T-CHO (mg/dl)**	**204.9 ± 36.1 (93–380)**	**211.6 ± 33.2 (96–329)**	**(<0.05)**
**TG (mg/dl)**	**176.8 ± 106.1 (36–1353)**	**123.0 ± 63.4 (29–689)**	**(<0.001)**
**HDL (mg/dl)**	**49.2 ± 10.9 (26–102)**	**59.6 ± 13.8 (33–127)**	**(<0.001)**
**LDL (mg/dl)**	**133.5 ± 32.2 (48–286)**	**135.5 ± 29.9 (48–243)**	**(NS)**
**Dyslipidemia, *n* (%)**	**788 (74.1%)**	**977 (93.3%)**	**(<0.001)**
**UA (mg/dl)**	**6.4 ± 1.4 (1–10.9)**	**4.9 ± 1.1 (1.4–8.9)**	**(<0.001)**
**FPG (mg/dl)**	**114.1 ± 22.5 (65–269)**	**108.1 ± 19.4 (78–272)**	**(<0.05)**
**HbA1c (%)**	**6.1 ± 0.9 (1.4–12.0)**	**6.1 ± 0.7 (4.8–11.7)**	**(NS)**
**IGT, *n* (%)**	**883 (83.1%)**	**777 (74.2%)**	**(NS)**
**ALT (IU/l)**	**41.0 ± 27.0 (7–210)**	**27.5 ± 19.8 (6–228)**	**(<0.001)**
**AST (IU/l)**	**28.3 ± 14.1 (9–139)**	**23.8 ± 11.0 (10–153)**	**(<0.001)**
**GGT (IU/l)**	**67.9 ± 66.1 (10–663)**	**33.8 ± 30.6 (7–424)**	**(<0.001)**
**FIB-4 index**	**1.2 ± 0.8 (0.24–13.7)**	**1.2 ± 0.5 (0.30–3.13)**	**(<0.005)**
**Fatty liver, *n* (%)**	**799 (75.2%)**	**602 (57.5%)**	**(<0.001)**
**V-type MS, *n* (%)**	**657 (61.8%)**	**154 (14.7%)**	**(<0.001)**
**Alcohol consumption, *n* (%)**	**784 (73.8%)**	**250 (23.9%)**	**<0.001**
**Past history of smoking, *n* (%)**	**771 (72.5%)**	**82 (7.8%)**	**<0.001**

ALT, alanine aminotransferase; AST, aspartate aminotransferase; BMI, body mass index; DBP, diastolic blood pressure; FPG, fasting plasma glucose; GGT, gamma-glutamyl transpeptidase; HbA1c, hemoglobin A1c; HDL, high density lipoprotein; IGT, impaired glucose tolerance; LDL, low density lipoprotein; MS, metabolic syndrome; NS, not significant; SBP, systolic blood pressure; T-CHO, total cholesterol; TG, triglyceride; UA, uric acid; V-type, visceral type; WC, waist circumference.

Data are given as means ± standard deviation (SD), and as number (%) for categorical variables. *P*-value is based on the χ^2^ test or Student’s t-test. Significant is at the 5% level.

^**a**^: Adjusted for age, BMI, WC, smoking history, hyperlipidemia, hypertension, and, IGT.

### Comparison of clinical characteristics in each MS type and each gender

As shown in Table 3, BMI and WC were significantly higher in V-type MS than S-type MS in both males and females with MS. (*P* <0.001). There was no significant difference in SBP, DBP, HDL, LDL, UA, FPG, and HbA1c between V-type MS and S-type MS in males with MS; however, TG, UA, FPG, and HbA1c in V-type MS was significantly higher in S-type MS in females with MS. The prevalence of FL with ALT elevation, ALT, AST, and GGT was significantly higher in V-type MS than in S-type MS in both males and females with MS. BMI and WC were significantly higher in males than females in both MS type (*p* <0.001). DBP, TG, and UA were significantly higher in males than females in both MS type (*p*^*a*^ <0.05). HDL was significantly higher in females than males in both MS type (*p*^*a*^ <0.001). The prevalence of FL with ALT elevation and ALT was significantly higher in males than females in V-type MS (*p*^*a*^ <0.01). The prevalence of alcohol consumption and past history of smoking was significantly higher in males than in females in both MS type (*p* <0.001).

**Table 3:** Comparison of clinical characteristics in each MS type and each gender.

	Males		Females		P-value (P-value^a^)			
	(1) V-type MS	(2) S-type MS	(3) V-type MS	(4) S-type MS	(1) vs (2)	(3) vs (4)	(1) vs (3)	(2) vs (4)
	(n = 657)	(n = 406)	(n = 154)	(n = 893)				
**Age (years)**	**52.2 ± 10.0**	**55.3 ± 8.8**	**52.7 ± 8.9**	**57.6 ± 8.0**	**<0.001**	**<0.001**	**NS**	**<0.001**
**BMI (kg/m**^**2**^**)**	**28.5 ± 3.4**	**27.7 ± 3.2**	**27.3 ± 3.7**	**26.1 ± 3.5**	**<0.001**	**<0.001**	**<0.001**	**<0.001**
**WC (cm)**	**96.9 ± 7.3**	**96.0 ± 6.7**	**91.5 ± 7.4**	**89.7 ± 7.1**	**<0.05**	**<0.01**	**<0.001**	**<0.001**
**SBP (mmHg)**	**129.1 ± 15.0**	**130.5 ± 15.1**	**130.4 ± 13.1**	**131.1 ± 14.4**	**(NS)**	**(NS)**	**(NS)**	**(NS)**
**DBP (mmHg)**	**79.9 ± 10.1**	**80.1 ± 10.3**	**79.1 ± 9.1**	**77.6 ± 9.6**	**(NS)**	**(NS)**	**(<0.05)**	**(<0.005)**
**T-CHO (mg/dl)**	**206.7 ± 36.4**	**202.0 ± 35.4**	**212.2 ± 27.7**	**211.5 ± 34.0**	**(NS)**	**(NS)**	**(NS)**	**(<0.01)**
**TG (mg/dl)**	**180.7 ± 98.9**	**170.6 ± 116.6**	**141.7 ± 59.9**	**119.8 ± 63.4**	**(NS)**	**(<0.001)**	**(<0.005)**	**(<0.001)**
**HDL (mg/dl)**	**48.6 ± 11.0**	**50.2 ± 10.7**	**56.3 ± 12.0**	**60.1 ± 14.0**	**(NS)**	**(NS)**	**(<0.001)**	**(<0.001)**
**LDL (mg/dl)**	**135.3 ± 32.7**	**130.6 ± 31.1**	**137.5 ± 25.4**	**135.2 ± 30.7**	**(NS)**	**(NS)**	**(NS)**	**(<0.05)**
**UA (mg/dl)**	**6.4 ± 1.4**	**6.3 ± 1.3**	**5.3 ± 1.1**	**4.9 ± 1.1**	**(NS)**	**(<0.001)**	**(<0.001)**	**(<0.001)**
**FPG (mg/dl)**	**115.0 ± 24.0**	**112.7 ± 19.7**	**113.0 ± 24.5**	**107.2 ± 18.3**	**(NS)**	**(<0.005)**	**(NS)**	**(NS)**
**HbA1c (%)**	**6.2 ± 0.9**	**6.1 ± 0.8**	**6.3 ± 1.0**	**6.0 ± 0.6**	**(NS)**	**(<0.05)**	**(NS)**	**(<0.001)**
**ALT (IU/l)**	**47.0 ± 28.0**	**31.2 ± 21.9**	**38.0 ± 24.2**	**25.7 ± 18.3**	**(<0.001)**	**(<0.001)**	**(<0.001)**	**(NS)**
**AST (IU/l)**	**30.7 ± 14.4**	**24.6 ± 12.6**	**28.9 ± 15.1**	**22.9 ± 9.9**	**(0.001)**	**(0.001)**	**(NS)**	**(NS)**
**GGT (IU/l)**	**73.3 ± 67.5**	**59.3 ± 62.9**	**42.8 ± 33.6**	**32.3 ± 29.8**	**(<0.005)**	**(<0.001)**	**(<0.005)**	**(<0.001)**
**FIB-4 index**	**1.1 ± 0.5**	**1.3 ± 1.1**	**1.1 ± 0.5**	**1.2 ± 0.5**	**(NS)**	**(NS)**	**(NS)**	**(NS)**
**FL with ALT elevation (+/-)**	**431/226 (65.6%)**	**97/309 (23.9%)**	**77/77 (50.0%)**	**160/733 (17.9%)**	**(<0.001)**	**(<0.001)**	**(<0.01)**	**(NS)**
**Alcohol consumption (+/-)**	**469/188 (71.4%)**	**315/91 (77.6%)**	**39/115 (25.3%)**	**211/682 (23.6%)**	**<0.05**	**NS**	**<0.001**	**<0.001**
**Past history of smoking (+/-)**	**468/189 (71.2%)**	**303/103 (74.6%)**	**8/146 (5.2%)**	**74/819 (8.3%)**	**NS**	**NS**	**<0.001**	**<0.001**

ALT, alanine aminotransferase; AST, aspartate aminotransferase; BMI, body mass index; DBP, diastolic blood pressure; FL, fatty liver; FPG, fasting plasma glucose; GGT, gamma-glutamyl transpeptidase; HbA1c, hemoglobin A1c; HDL, high-density lipoprotein; LDL, low-density lipoprotein; MS, metabolic syndrome; NS, not significant; SBP, systolic blood pressure; S-type, subcutaneous type; T-CHO, total cholesterol; TG, triglyceride; UA, uric acid; V-type, visceral type; WC, waist circumference. ALT elevation was defined as ALT level of ≥31 IU/l in this study.

Data represent the means ± standard deviation (SD), and number (%) for categorical variables.

*P*-value is based on the χ^2^ test or Student’s t-test. Significant is at the 5% level.

^**a**^: Adjusted for age, BMI, WC, smoking history, hyperlipidemia, hypertension, and impaired glucose tolerance.

### Independent predictors of FL with ALT elevation in males and females with MS

Table 4 shows the univariate and multivariate analyses for independent predictors of FL with ALT elevation in males and females with MS. Of the 20 items related to the clinical background of the subjects who had FL with ALT elevation or the subjects who did not have FL with ALT elevation, 15 and 14 items were identified as significant factors by univariate analysis in males and females with MS, respectively. In males with MS, multiple logistic regression analysis was performed with 9 items, with age, BMI, WC, hypertension, dyslipidemia, UA, HbA1c, MS type, and status of drinking as covariates. Age, WC, dyslipidemia, HbA1c, and V-type MS were significant and independent predictors of an increased prevalence of FL with ALT elevation. Light drinker was a significant and independent predictor of a decreased prevalence of FL with ALT elevation. The OR (95% CI, *p* value) for FL with ALT elevation were as follows: age, 2.874 (2.110–3.915, *p* <0.001); WC, 1.831 (1.360–2.464, *p* <0.005); dyslipidemia, 1.893 (1.339–2.676, *p* <0.005); HbA1c, 1.360 (1.003–1.845, *p* <0.05); V-type MS, 5.783 (4.288–7.799, *p* <0.001); and light drinker, 0.563 (0.407–0.778, *p* <0.005). On the other hands, in females with MS, multiple logistic regression analysis was performed with 10 items, with age, BMI, WC, SBP, DBP, TG, HDL-C, UA, IGT, and MS type as covariates. BMI, WC, DBP, TG, UA, IGT, and V-type MS were significant and independent predictors of an increased prevalence of FL with ALT elevation. The OR (95% CI, *p* value) for FL with ALT elevation were as follows: BMI, 2.179 (1.425–3.332, *p* <0.001); WC, 1.850 (1.267–2.702, *p* <0.005); DBP, 1.687 (1.163–2.449, *p* <0.01); TG, 2.221 (1.558–3.166, *p* <0.001); UA, 2.911 (1.419–5.969, *p* <0.01); IGT, 1.658 (1.112–2.471, *p* <0.05); and V-type MS, 3.828 (2.571–5.700, *p* <0.001).

**Table 4:** Results of univariate and multivariate analyses: Independent predictors of fatty liver with ALT elevation in males and females with MS.

	Males with MS (n = 1063				Females with MS (n = 1047			
	Univariate analysis	Multivariate analysis			Univariate analysis	Multivariate analysis		
	*P*-value	OR	95% CI	*P*-value	*P*-value	OR	95% CI	*P*-value
**Age (< 50/≧ 50 years)**	**<0.001**	**2.874**	**2.110–3.915**	**<0.001**	**<0.005**	**1.007**	**0.672–1.509**	**NS**
**BMI (≧ 25/< 25 kg/m**^**2**^**)**	**<0.001**	**1.497**	**0.933–2.403**	**NS**	**<0.001**	**2.179**	**1.425–3.332**	**<0.001**
**WC (≧ 95 or 90/< 95 or 90 cm)**	**<0.001**	**1.831**	**1.360–2.464**	**<0.005**	**<0.001**	**1.850**	**1.267–2.702**	**<0.005**
**SBP (≧ 130/< 130 mmHg)**	**NS**				**<0.05**	**0.711**	**0.506–1.000**	**NS**
**DBP (≧ 85/< 85 mmHg)**	**NS**				**<0.05**	**1.687**	**1.163–2.449**	**<0.01**
**Hypertension (+/-)**	**<0.005**	**0.970**	**0.684–1.375**	**NS**				
**T-CHO (≧ 220/< 220 mg/dl)**	**<0.005**			**NS**				
**TG (≧ 150/< 150 mg/dl)**	**<0.001**				**<0.001**	**2.221**	**1.558–3.166**	**<0.001**
**HDL (< 40 or 50/≧40 or 50 mg/dl)**	**<0.05**				**<0.001**	**1.334**	**0.929–1.915**	**NS**
**LDL (≧ 140/< 140 mg/dl)**	**<0.001**				**NS**			
**Dyslipidemia (+/-)**	**<0.001**	**1.893**	**1.339–2.676**	**<0.005**	**NS**			
**UA (≧ 7.0/< 7.0 mg/dl)**	**<0.005**	**1.238**	**0.907–1.689**	**NS**	**<0.001**	**2.911**	**1.419–5.969**	**<0.01**
**FPG (≧ 100/< 100 mg/dl)**	**NS**				**<0.05**			
**HbA1c (≧ 6.2/< 6.2%)**	**<0.05**	**1.360**	**1.003–1.845**	**<0.05**	**<0.001**			
**IGT (+/-)**	**NS**				**<0.05**	**1.658**	**1.112–2.471**	**<0.05**
**AST (≧ 31/< 31 IU/l)**	**<0.001**				**<0.001**			
**GGT (≧ 51/< 51 IU/l)**	**<0.001**				**<0.001**			
**V-type MS / S-type MS**	**<0.001**	**5.783**	**4.288–7.799**	**<0.001**	**<0.001**	**3.828**	**2.571–5.700**	**<0.001**
**Light drinker/ Non-drinker**	**<0.001**	**0.563**	**0.407–0.778**	**<0.005**	**NS**			
**Past history of smoking (+/-)**	**NS**				**NS**			

ALT, alanine aminotransferase; AST, aspartate aminotransferase; BMI, body mass index; CI, confidence interval; DBP, diastolic blood pressure; FL, fatty liver; FPG, fasting plasma glucose; GGT, gamma-glutamyl transpeptidase; HbA1c, hemoglobin A1c; HDL, high-density lipoprotein; IGT, impaired glucose tolerance; LDL, low-density lipoprotein; MS: metabolic syndrome; NS, not significant; OR, odds ratio; SBP, systolic blood pressure; S-type, subcutaneous type. T-CHO, total cholesterol; TG, triglyceride; UA, uric acid; V-type, visceral type; WC, waist circumference. Male subjects who drank from more than zero but less than 20 g/day of alcohol were defined as light drinkers. Male non-drinkers were subjects who drank 12 drinks or less per year of less than 20 g/drinking day. Female subjects who drank from more than zero but less than 10 g/day of alcohol were defined as light drinkers. Female non-drinkers were subjects who drank 12 drinks or less per year of less than 10 g/drinking day. FL with ALT elevation was referred to as FL with ALT level of ≥31 IU/l in the present study.

Significant is at the 5% level.

### The influence of the treatment for lifestyle-related diseases and lifestyle on the prevalence of FL with ALT elevation in males and females with MS

As shown in Table 5, although there was no significant difference in the prevalence of those receiving treatment for hypertension between genders with MS, the prevalence of FL with ALT elevation were significantly higher in males with MS than in females with MS in both those with and without treatment for hypertension. The prevalence of those receiving treatment for dyslipidemia was significantly higher in females with MS than in males with MS. Additionally, the prevalence of FL with ALT elevation were significantly higher in males with MS than in females with MS in both those with and without treatment for dyslipidemia. Although the prevalence of those receiving treatment for IGT was significantly higher in males with MS than in females with MS, there was no significant difference in the prevalence of FL with ALT elevation between genders with MS in those receiving treatment for IGT. However, the prevalence of FL with ALT elevation was significantly higher in males with MS than in females with MS in those without treatment for IGT. The prevalence of those with a past history of smoking and light alcohol consumption (LAC) was significantly higher in males with MS than in females with MS. The prevalence of FL with ALT elevation was significantly higher in males with MS than in females with MS in both those with and without a past history of smoking or LAC.

**Table 5:** Comparison of the influence of the treatment for lifestyle-related diseases and lifestyle on the prevalence of FL with ALT elevation between males and females with MS.

Lifestyle-related disease or lifestyle	Presence of treatment or favorite item	Males with MS (n = 1063)	Females with MS (n = 1047)	P-value(P^a^-value)
Hypertension	Treatment (+), n (%)	400 (37.6%)	419 (40.0%)	NS
	FL with ALT elevation, n (%)	164 (41.0%)	108 (25.8%)	(<0.005)
	Treatment (-), n (%)	663 (62.4%)	628 (60.0%)	
	FL with ALT elevation, n (%)	364 (54.9%)	129 (20.5%)	(<0.001)
Dyslipidemia	Treatment (+), n (%)	226 (21.3%)	387 (37.0%)	<0.0001
	FL with ALT elevation, n (%)	117 (51.8%)	90 (23.3%)	(<0.005)
	Treatment (-), n (%)	837 (78.7%)	660 (63.0%)	
	FL with ALT elevation, n (%)	411 (49.1%)	147 (22.3%)	(<0.001)
IGT	Treatment (+), n (%)	139 (13.1%)	84 (8.0%)	<0.001
	FL with ALT elevation, n (%)	65 (6.1%)	28 (2.7%)	(NS)
	Treatment (-), n (%)	924 (86.9%)	963 (92.0%)	
	FL with ALT elevation, n (%)	463 (50.1%)	209 (21.7%)	(<0.001)
Past history of smoking	smoking (+), n (%)	771 (72.5%)	82 (7.8%)	<0.001
	FL with ALT elevation, n (%)	369 (47.9%)	24 (29.3%)	(<0.001)
	smoking (-), n (%)	292 (27.5%)	965 (92.2%)	
	FL with ALT elevation, n (%)	159 (54.5%)	213 (22.1%)	(<0.001)
LAC	LAC (+), n (%)	784 (73.8%)	250 (23.9%)	<0.001
	FL with ALT elevation, n (%)	357 (45.5%)	60 (24.0%)	(<0.001)
	LAC (-), n (%)	279 (26.2%)	797 (76.1%)	
	FL with ALT elevation, n (%)	171 (61.3%)	177 (22.2%)	(<0.001)

ALT, alanine aminotransferase; FL, fatty liver; IGT, impaired glucose tolerance; LAC, light alcohol consumption; MS, metabolic syndrome; NS, not significant. Male subjects who drank from more than zero but less than 20 g/day of alcohol were defined as positive for LAC. Female subjects who drank from more than zero but less than 10 g/day of alcohol were defined as positive for LAC.

Data represent the means ± standard deviation (SD), and number (%) for categorical variables.

*P*-value is based on the χ^2^ test or Student’s t-test. Significant is at the 5% level.

^**a**^: Adjusted for age, BMI, WC, hyperlipidemia, hypertension, IGT, smoking history, and LAC.

## Discussion

The aim of the present study was to compare the clinical backgrounds of males and females with MS, and to explore the differing influence of lifestyle-related factors, and visceral fat on FL with ALT elevation between genders with MS. Although MS has been known to be strongly associated with NAFLD in both sexes, the prevalence of MS and NAFLD is different between genders [8, 9]. Although there are some reports of an association between NAFLD and clinical backgrounds in individuals with MS [10, 11], the differing influence of lifestyle-related factors and visceral fat on FL with elevation of liver enzymes between genders with MS is unclear. The present study showed that V-type MS is a common significant predictor of an increased prevalence of FL with ALT elevation in both sexes with MS, however, there were several different lifestyle-related factors for the prevalence of FL with ALT elevation between genders with MS.

While males with MS and females with MS have been treated in the same manner to date, we found several differences in physical constitution between genders with MS. Although the visceral fat area is larger in individuals with MS than in those with non-applicable MS, we noticed that there may be a difference in the proportion of visceral fat even in the same individuals with MS, and also, there may be a difference in the distribution of fat accumulation between genders with MS. The proportion of V-type MS and S-type MS was 38.4% and 61.6%, respectively in present study; these results suggesting that, although all subjects were diagnosed with MS, they may not necessary have visceral fat-dominant MS. In addition, there was a considerable discrepancy in the proportion of MS types between genders with MS, in other words, the proportion of V-type MS (61.8%) was significantly higher than that of S-type MS (38.2%) in males with MS, and S-type MS (85.3%) was significantly higher than V-type MS (14.7%) in females with MS. Females are more likely to develop peripheral adiposity with gluteal and subcutaneous fat accumulation, whereas males are more prone to central fat accumulation [12, 13]. Although the mechanism underlying the differences in body fat distribution between genders are not yet clear, sex hormones may play a major role in determining body fat distribution [14–17]. For example, body fat distribution changes with shifts in sex hormones during puberty or menopause [14]. Sex steroids decrease with age and induce a shift in regional body fat distribution towards a more central fat distribution in both sexes [15, 16]. The difference of body fat distribution and the proportion of visceral fat between genders or MS types may be influenced partially by the change and the balance of sex hormones.

Additionally, we noticed that clinical backgrounds might be different between individuals with V-type MS and S-type MS, even if they were defined as same MS. With regard to physical constitution, BMI and WC were significantly higher in individuals with V-type MS than in those with S-type MS in both sexes. With regard to factors associated with lifestyle-related diseases, TG, FPG, HbA1c, and UA were significantly higher in females with V-type MS than in those with S-type MS. With regard to the influence on the liver, the prevalence of FL with ALT elevation, ALT, and AST were significantly higher in individuals with V-type MS than in those with S-type MS in both sexes. V-type MS may be more influenced by various clinical backgrounds, compared to S-type MS. Visceral fat and subcutaneous fat are both considered to be central features of MS and, in particular, visceral adipose tissue is thought to be a source of inflammatory cytokines and to be associated with systemic inflammation in obese individuals [18, 19]. Tilg et al. proposed that adipocytes and infiltrating macrophages in visceral adipose tissue produced a large amount of systemically active mediators, including adipocytokines, thought to contribute to low-grade inflammation observed in severe obesity, MS, and other associated disorders [20]. Visceral fat accumulation was reported to play an important role in the development of NAFLD [10, 21, 22]. Elevation of serum ALT is closely associated with liver fat content and NAFLD, especially in populations with a very high prevalence of obesity and FL in several epidemiological reports [11]. Moreover, we surmised that there might be several different clinical characteristics between genders with each MS type. Several factors associated with lifestyle-related diseases and the proportion of FL with ALT elevation were significantly higher in males with V-type MS than in females with V-type MS. On the other hand, although there were some different clinical characteristics between genders with S-type MS, there was no significantly difference in the prevalence of FL with ALT elevation between genders with S-type MS. These findings suggest that differences in MS type and gender should be considered for FL with ALT elevation in individuals with MS.

It is known that improvement of lifestyle-related diseases is important for individuals with MS or NAFLD in both sexes. The present study showed that the prevalence of lifestyle-related diseases and the influence of lifestyle-related diseases on FL with ALT elevation were different between genders with MS; however, IGT and dyslipidemia were common important factors for the presence of FL with ALT elevation in both sexes with MS. Although the cause of NAFLD is not yet fully understood, in individuals with MS with an increased rate of lipid ingestion and amount of visceral adipose tissue, free fatty acids produced by neutral fat degradation are increased and flow into the liver via the portal vein [23–25], potentially causing lipotoxicity and hepatocyte inflammation. Insulin resistance that has a possibility of leading to fat accumulation in hepatocytes by lipolysis and hyperinsulinemia may be highly associated with NASH [26, 27].

Lifestyle, such as smoking and alcohol consumption, is known to influence NAFLD. Exact pathogenetic roles of the association between smoking and NAFLD and the mechanisms of smoking-induced fatty changes and fibrosis in the liver are still unclear. Oxidative stress is known to be a mechanism of injury in NAFLD, and cigarette smoking is thought to be an inducer of oxidative stress and have pro-inflammatory effects in other organs [28–30]. In the present study, smoking history was not a significant predictor of FL with ALT elevation in both sexes with MS. Heavy drinking is known to cause liver injury [31], and light or moderate drinking has been reported to play a protective role against FL in recent studies [32, 33]. Although the influence of alcohol consumption is known to be markedly different between genders [34–36], the association between alcohol consumption, including drinking styles and volume, and NAFLD has remained controversial. Interestingly, although LAC was a significant predictor of a decreased prevalence of FL with ALT elevation in males with MS, LAC was not a significant predictor in females with MS. The present findings suggest that males may differ from females in sensitivity to the hepatic effects of alcohol due to the pharmacokinetics or metabolism of alcohol [35]. Although the mechanism of the association between LAC and FL with ALT elevation is unclear, some studies have reported that moderate alcohol intake enhances insulin sensitivity [37]. LAC may reduce the risk of NASH due to reduced insulin resistance [32].

Elevation of age has known to be a risk factor for NAFLD in females, but in males. However, this study showed that age of females with MS was not a significant risk factor for fatty liver with ALT elevation in multivariate analyses. Generally, the prevalence of NAFLD in premenopausal females has known to be lower than in postmenopausal females. These previous results considered to be related to the state of menses. In this study, the prevalence of alcohol consumption in females under 50 years of age who had MS was significantly higher than in females of 50 years and upward who had MS (data not shown). In addition, the prevalence of past history of smoking in persons under 50 years of age and persons of 50 years and upward was 10.9% and 7.1%, in females with MS, respectively (data not shown). Female results in this study may be more influenced by lifestyle factors, such as drinking and smoking than age and the state of menses.

The present study showed that the predictors for FL with ALT elevation in males with MS were partly different from those in females with MS. However, we suggest that it is desirable for individuals with MS to improve WC and V-type MS, because WC and V-type MS were common predictors of an increased prevalence of FL with ALT elevation. Additionally, while the influence of lifestyle-related diseases on FL with ALT elevation was different between genders with MS, we suggest that it is important for individuals with MS to improve lifestyle-related diseases such as dyslipidemia and IGT, because the factors associated with dyslipidemia and IGT were significantly related to FL with ALT elevation. With regard to lifestyle, while smoking habit was not a significant predictor of an increased prevalence of FL with ALT elevation, smoking should not be recommended for non-smokers, as cigarette smoking is known to be strongly associated with lung cancer in various countries [38]. Although LAC was a significant predictor of a decreased prevalence of FL with ALT elevation in males with MS, we should not suggest that drinking is recommended for non-drinkers, as the effects of drinking differ among individuals and genders; moreover, lifestyle-related diseases may be affected by increased eating and the increased calorie intake due to alcohol consumption.

There are several limitations to the present study. First, there was a possible selection bias, because most of the participants were healthy individuals who hoped to undergo a medical check-up. Second, detailed information of diets, such as volume and contents including vegetable and fruits, the duration of eating and drinking, and total caloric intake, were not investigated. Third, the state of menses in females was not investigated, and the levels of estrogen-related sex hormones in both sexes were not measured. Last, the differences between ethnicities could not be assessed because all subjects were only Japanese.

## Conclusions

The present study showed that WC and V-type MS are common significant predictors of an increased prevalence of FL with ALT elevation in both sexes with MS. However, there were several different factors associated with lifestyle and lifestyle-related diseases between males and females with MS that influences the prevalence of FL with ALT elevation. Gender, lifestyle-related factors, and MS type in individuals with MS may contribute to the development of FL with ALT elevation.

## Supporting information

S1 Appendix The data set. The data provided in this article are available within this file.
(XLSX)

S1 File STROBE checklist. (PDF)
